# Supplementary material for: Material-specific high-resolution table-top extreme ultraviolet microscopy
Source: Light Sci Appl. 2022 Apr 29;11:117. doi: 10.1038/s41377-022-00797-6 (PMC9054792; doi:10.1038/s41377-022-00797-6)
Supplement: Supplementary file 1 — Supplement information [file 41377_2022_797_MOESM1_ESM.docx]

**Supplementary Information for:**

**Material-specific high-resolution table-top extreme ultraviolet microscopy**

*Wilhelm Eschen, Lars Loetgering, Vittoria Schuster, Robert Klas, Alexander Kirsche, Lutz Berthold, Michael Steinert, Thomas Pertsch, Herbert Gross, Michael Krause, Jens Limpert & Jan Rothhardt*

**Supplementary Note 1: Ptychography setup – optical design**

Here we present optical design considerations of the EUV mirror system regarding spectral filtering properties and optical aberrations. To spectrally filter the broadband EUV radiation at 13.5 nm three Mo/Si multilayer mirrors (optiX fab GmbH) are used. The mirrors were coated in a single run. After fabrication, the reflectivity of the multilayer was characterized at the Physikalisch-Technische Bundesanstalt (PTB) yielding a reflectivity of more than 60% and a bandwidth (FWHM) of 0.4 nm at a 4° incidence angle. The varying incidence angles of up to 10° on the EUV mirrors lead to additional spectral shifts, which were already accounted for during the design process. Altogether, the three multilayer mirrors yield a total reflectivity of 19% and a spectral bandwidth (FWHM) of 0.2 nm at 13.5 nm central wavelength. To achieve a small focal spot size, a three-mirror out-of-plane configuration was chosen. The basic idea of the more complicated 3D geometry of the curved mirror system is to correct the primary astigmatism according to the well-known principle of the Schiefspiegler telescope. The remaining aberrations are coma and second-order astigmatism, which are one order of magnitude smaller. Therefore the focus quality can be improved considerably and the number of mirrors remains small to avoid a decreased useable photon flux. Furthermore, the use of spherical mirrors guarantees components with high-quality surfaces and avoids expensive toric mirrors, which are fabricated by diamond turning and usually suffer from mid-spatial frequency errors^1^. The top and side view schematics of the setup are shown in Figure S1. The first mirror (M1) is a planar mirror that steers the beam by 9.9° to the side (around the y axis) and by 4.6° down (around the x-axis). This mirror has no effect on optical performance but has been introduced in order to keep the focused output beam in the same horizontal plane as the input beam. The second (M2) and third mirror (M3) are spherical mirrors. Their focal lengths have been chosen to image the HHG source onto the sample position in the vicinity of the camera flange, while the incidence angles are matched to compensate for astigmatism. M2 has a radius of curvature of 3000 mm and is intended to collimate the divergent EUV beam originating from the HHG source located at a distance of about 1500 mm from M2. M2 steers the beam by 10° around the y-axis; M3 (focal length 300mm) steers the EUV beam by 4.5° around the x-axis and focuses close to the sample.

The resulting EUV focus was characterized by a ptychography measurement and is shown in Figure S1 **c**. Since the characterization has been performed 600 µm downstream of the focus, the beam was numerically back-propagated into the focal plane. The resulting complex field is shown in Figure S1 **d**. Horizontal and vertical lineouts of the intensity yield a width of 3.8  µm and 2.6 µm (FWHM), respectively.


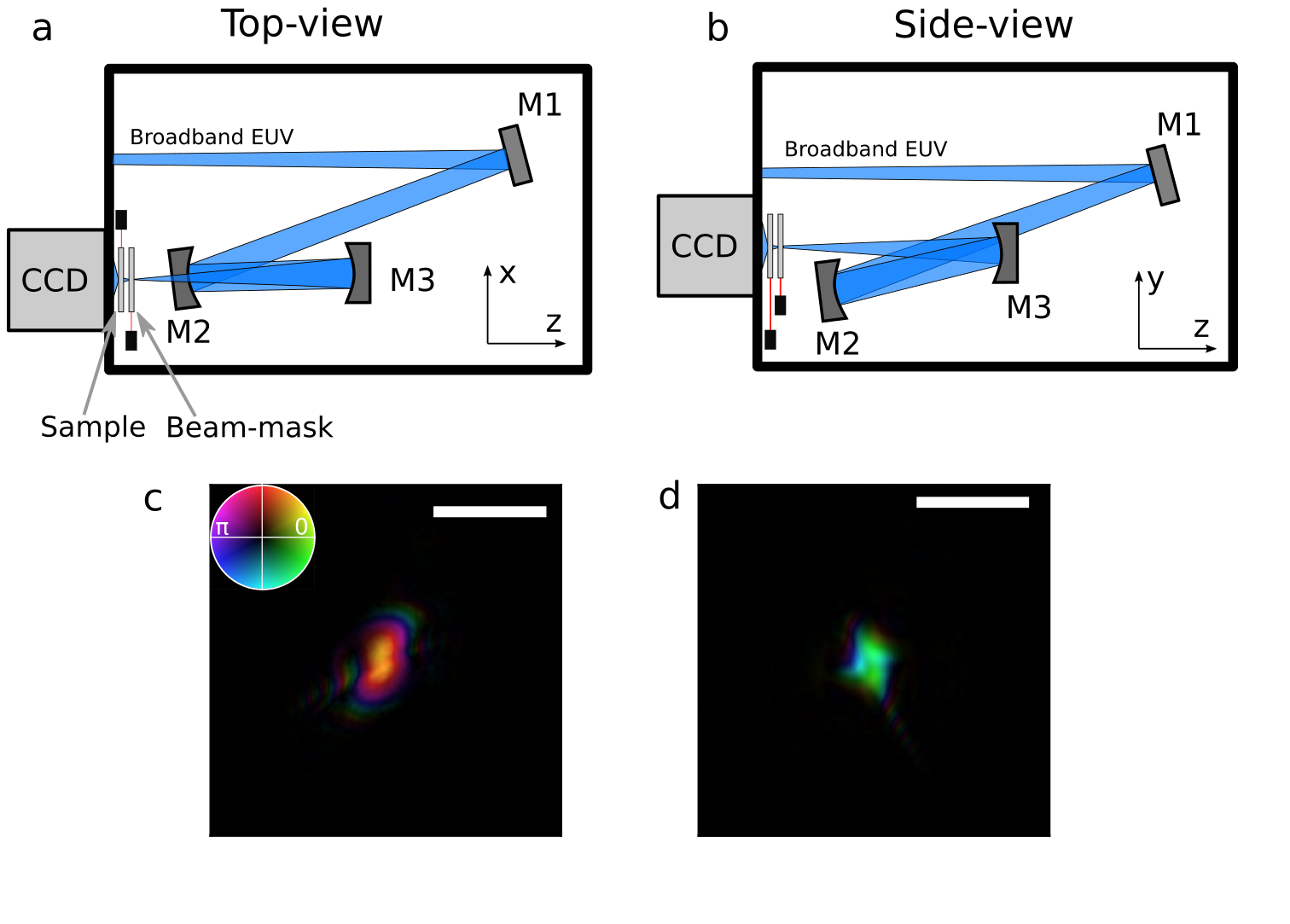


Figure S1: EUV ptychographic imaging setup and characterization of the focal spot. a Top-and b side-view of the imaging setup. c Reconstructed EUV beam at sample position (600 µm downstream of the focal plane). d Reconstructed EUV beam numerically back-propagated into the focal plane. The scale bars in c and d correspond to 10 µm.

**Supplementary Note 2: Resolution estimation of the integrated structure**

To quantify the achieved lateral resolution of the integrated circuit ptychographic reconstruction (Figure S2 **a**), a second identical measurement was performed and the Fourier ring correlation (FRC) was computed from both measurements (Figure S2 **d**). The application of the half-bit criterion yields a half-pitch resolution of 52 nm. Supplementary Figures 2 **b** shows a magnified view of the integrated circuit. A lineout along the white line in b is shown in c, indicating features with a half-pitch distance of 78 nm.


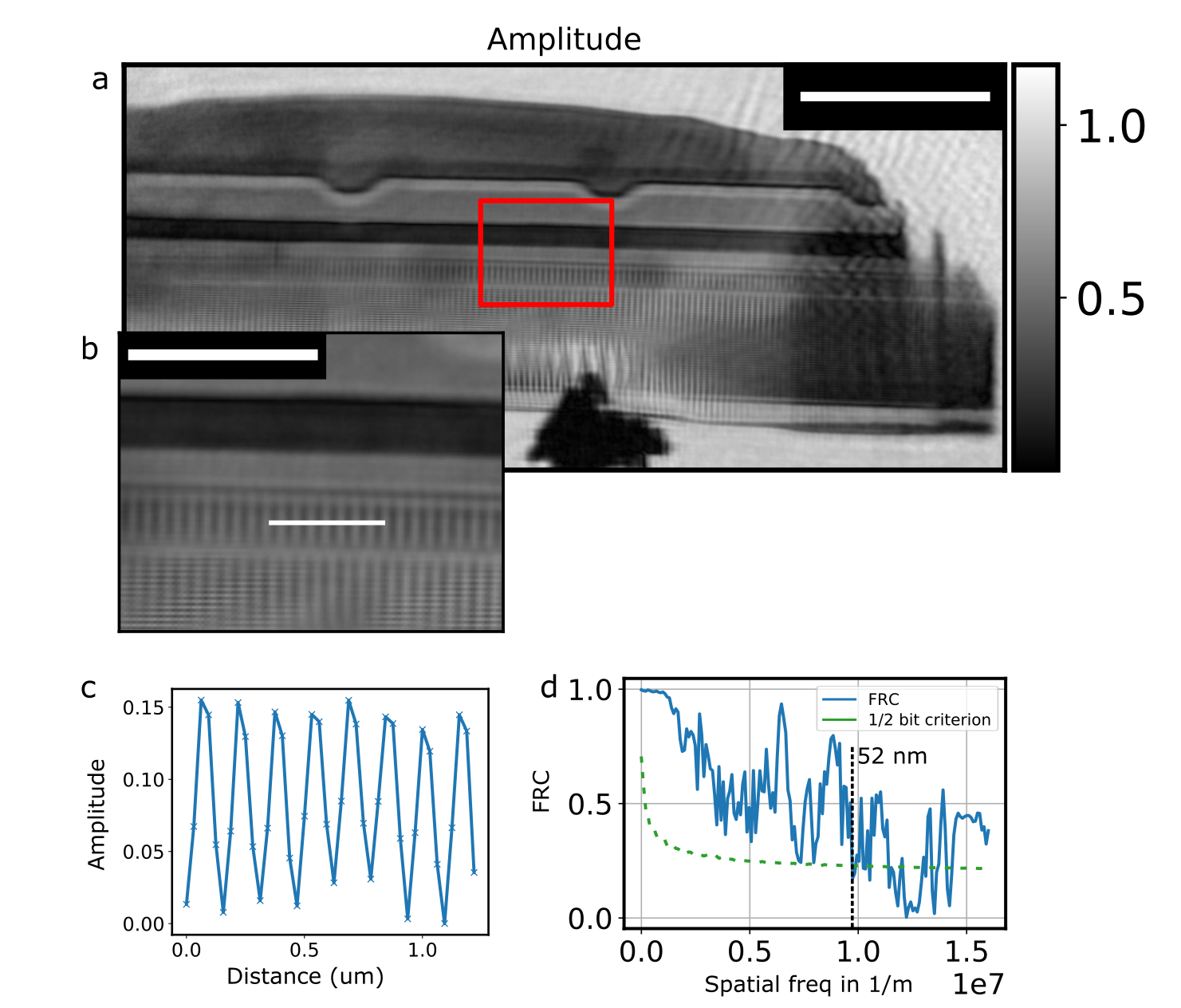


Figure S2: The reconstructed amplitude of the integrated structure is shown in a. b magnified view of the red box in a. c shows a lineout along the white line in b, indicating features with a half-pitch distance of 78 nm resolved. The Fourier ring correlation is shown in d and results in a half-pitch lateral resolution of 52 nm using the half-bit criterion. The white scale bars in a and b have a width of 5 µm and 2 µm, respectively.

**Supplementary Note 3: Mixed state ptychography**

For the Siemens star ptychography reconstructions presented in the manuscript, five mutually incoherent probe modes (mixed states) - which henceforth are referred to as *modes* here for brevity – are shown. Figure S3 **a** – **e** displays the modes that belong to the data set in Figure 3 of the main text. Each mode was back-propagated into the mask 200 µm upstream of the specimen. The main mode (**a,** Mode 0) contains approximately 60% of the power. Since HHG sources usually provide a high degree of spatial coherence^2^, we attribute the m-s modes to other sources of decoherence^3^, as mentioned in the Methods section of the main text.


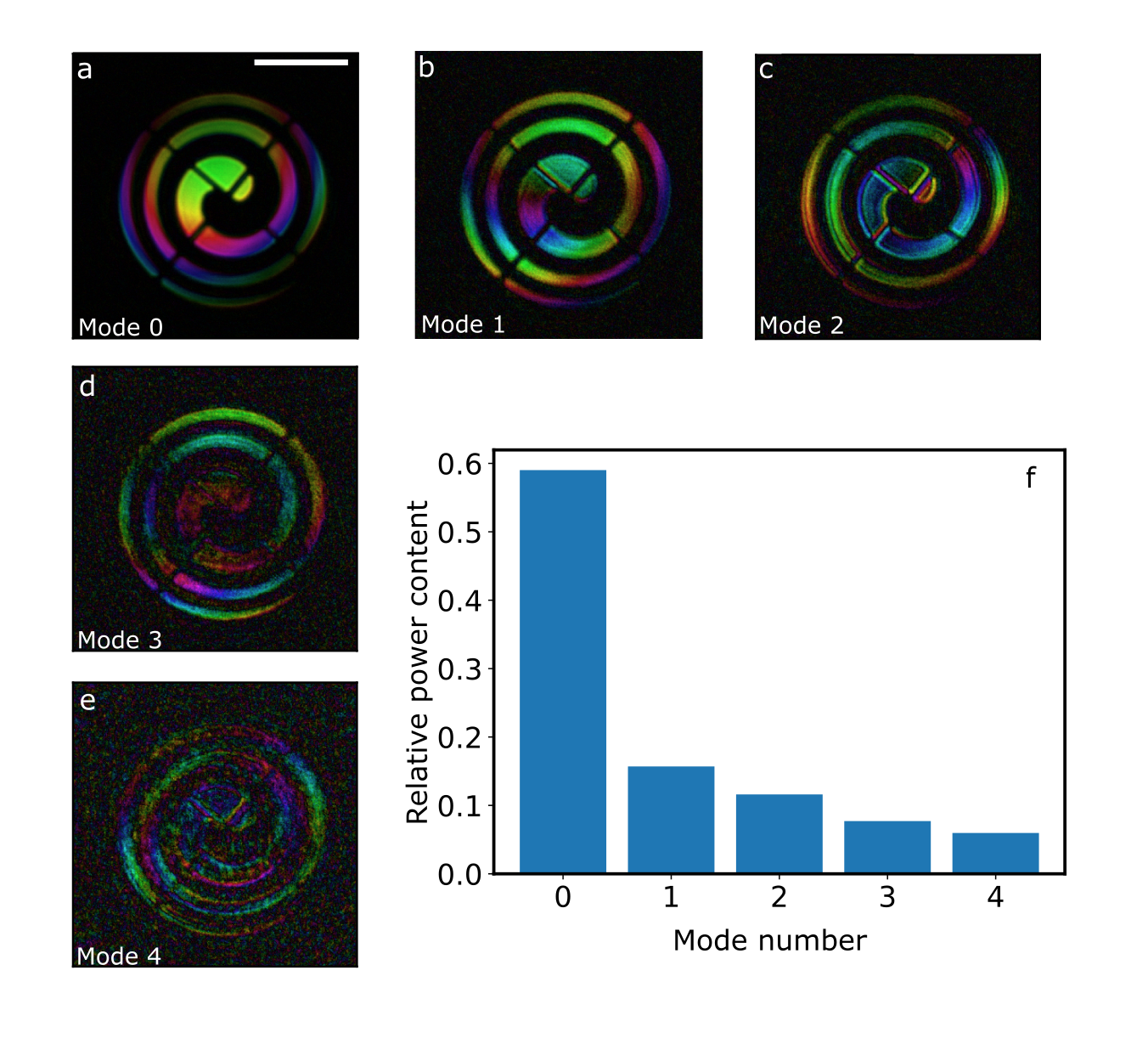


Figure S3: The reconstructed probe mixed states (modes) from the data set shown in Figure 3 in the main text are shown in a - e. The relative power content of the modes is shown in f. The scale-bar from a applies to the plots a-e and has a size of 5 µm.

**Supplementary Note 4: Mixed state orthogonal probe relaxation**

The orthogonal probe relaxation (OPR)^4^ method allows the reconstruction of probe modes that slightly change during the ptychography scan. Here we applied OPR to all m-s modes in order to maximize the quality of the reconstruction. To keep the memory footprint low, only 4 incoherent modes were used, each of which is synthesized from a linear combination of 4 OPR modes with scan-position-variant expansion coefficients. The resulting m-s OPR modes were back-propagated 550 µm into the mask plane and are shown in Figure S4. Here the OPR modes are arranged along the rows and m-s modes (mixed states) are shown along with the columns of the four-by-four matrix of modes. The main mode (m-s mode 0, OPR mode 0) shows the charge-1 OAM mask and a phase curvature which is due to the out-of-focus position of the mask. The OPR modes with power mostly contained in the support region of the mask (e.g. m-s mode 0, OPR mode 2) can be attributed to pointing instability of the EUV beam.


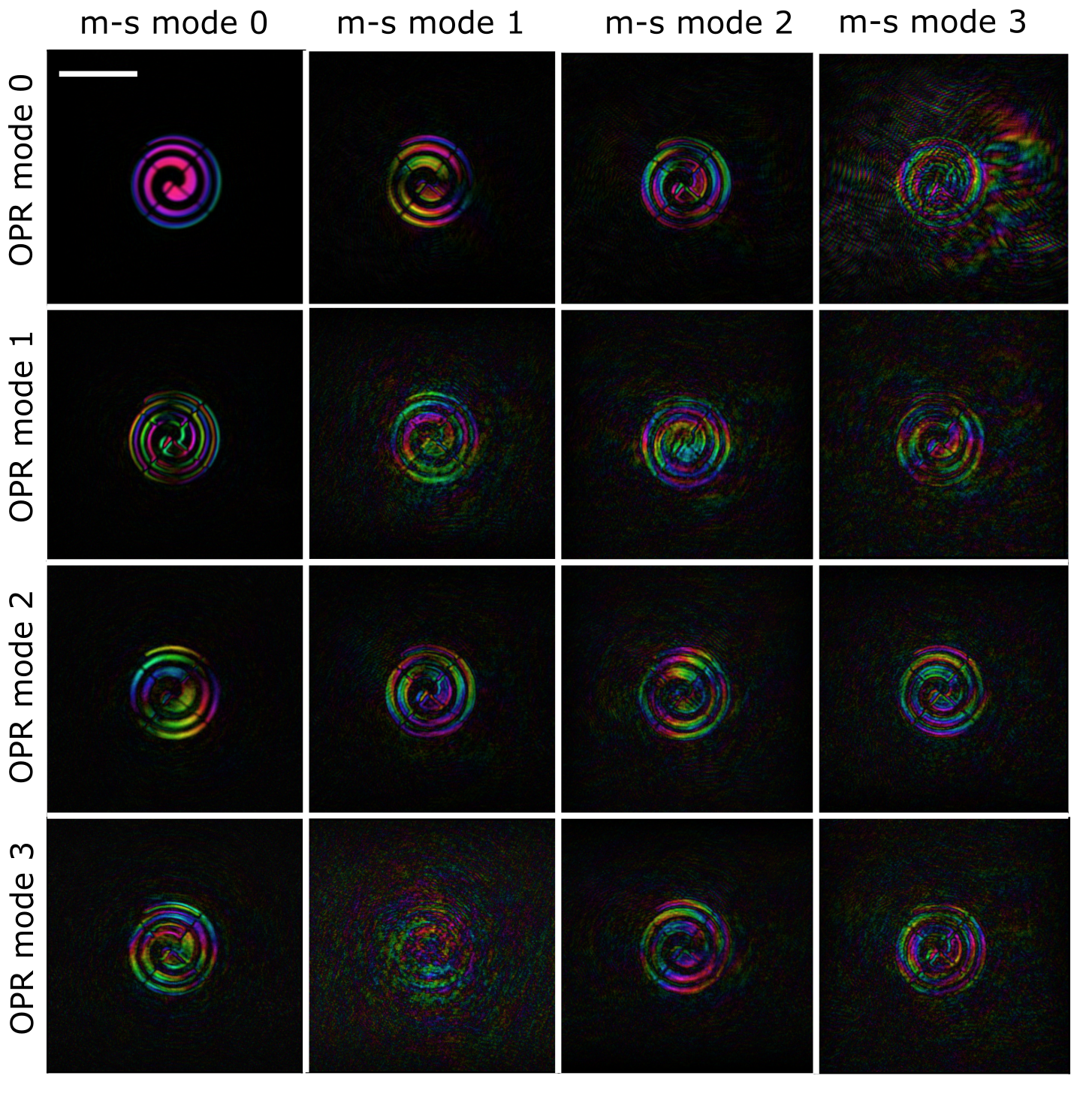


Figure S4: Overview of jointly retrieved OPR and mixed state modes, arranged along rows and colums. The white scale bar has a size of 10 µm.

The m-s OPR method provides the slowly evolving temporal variation of the m-s modes throughout the scan. Figure S5 a shows the m-s modes in the mask plane for the first position (position 0) and last position (position 134). Differences in the m-s mode structure for the first and last position are highlighted by the vertical phase-lineout shown in Figure S5 **b** and **c**. The change in the phase can be explained by slow drifts of the EUV-beam during the measurement.


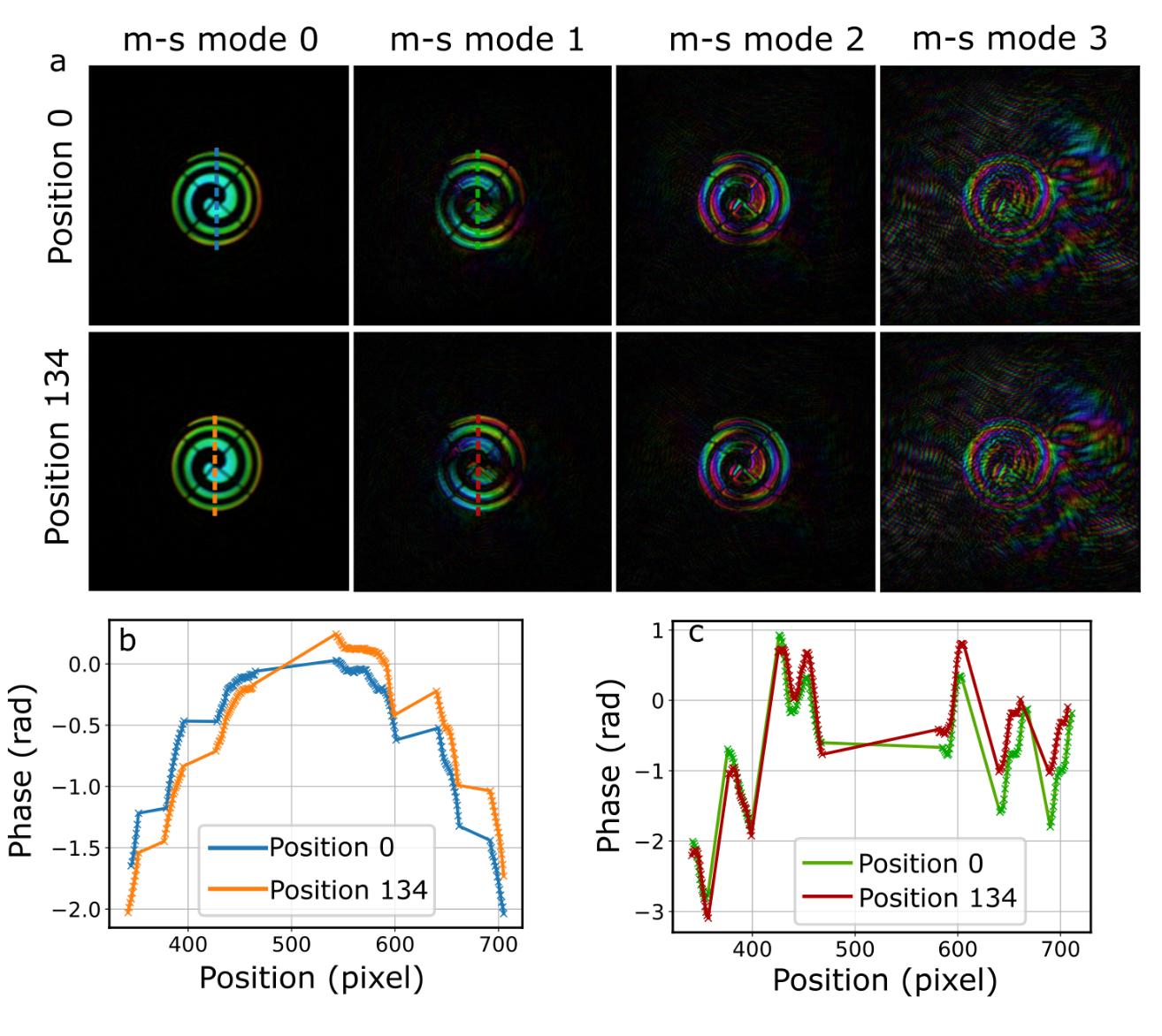


Figure S5: a shows the reconstructed m-s modes for the first position and the last position of the scan. The vertical phase lineouts for m-s mode 0 and m-s mode 1 are shown in b and c respectively. It is apparent, that the phase profile of the beam drifted with respect to the mask during the measurement, which can be attributed to a slow drift of the EUV beam pointing.

**Supplementary Note 5: Measurement overview**

| Measurement | # positions | scan step [µm] | on-chip binning | exposure time [s] | Total measurement time [min] | mask-sample distance | charge |
| --- | --- | --- | --- | --- | --- | --- | --- |
| OAM Fig. 2 | 101 | 1.0 | 2x2 | 5, 30 | 68 | 210 µm | 3 |
| Pinhole Fig. 2 | 101 | 1.0 | 2x2 | 2.5, 15 | 38 | 170 µm | 0 |
| Figure 3 | 101 | 1.0 | 2x2 | 3, 45 | 91 | 200 µm | 1 |
| Integrated circuit | 135 | 0.7 | 1x1 | 0.3, 2.5, 10 | 70 | 550 µm | 1 |

Table 1 Summary of parameters of the ptychography scans reported here. ‘# positions’ – total number of positions of the ptychography scan. ‘scan step [µm]’ – average distance between adjacent scan positions. ‘on-chip binning’ – on-chip binning that was used during the measurement. 2x2 on-chip binning results in an effective pixel area of 27 µm x 27 µm. ‘exposure time’ – CCD exposure times for each position. Multiple exposure times are used for HDR fusion of the diffraction data. ‘Total measurement time [min]’ – Required time for the whole measurement including the accumulated exposure time, read-out of the CCD and movement of the sample. ‘Mask-sample distance’ – distance between the mask and the sample. ‘charge’ – OAM induced by mask (azimuthal phase shift divided by 2π).

**References:**

1.

Fang, F. Z., Zhang, X. D., Weckenmann, A., Zhang, G. X. & Evans, C. Manufacturing and measurement of freeform optics. *CIRP Ann. - Manuf. Technol.* **62**, 823–846 (2013).

2. Bartels, R. A. *et al.* Generation of Spatially Coherent Light at Extreme Ultraviolet Wavelengths. *Science (80-. ).* **297**, 376–378 (2002).

3. Thibault, P. & Menzel, A. Reconstructing state mixtures from diffraction measurements. *Nature* **494**, 68–71 (2013).

4. Odstrcil, M. *et al.* Ptychographic coherent diffractive imaging with orthogonal probe relaxation. *Opt. Express* **24**, 8360 (2016)
